# Initial efforts to improve medical student information-seeking behavior with embedded library instruction

**DOI:** 10.5195/jmla.2023.1771

**Published:** 2023-10-02

**Authors:** Angela Barr

**Affiliations:** 1 ab3538@georgetown.edu, Reference and Digital Information Services Coordinator, Dahlgren Memorial Library Georgetown University School of Medicine, Washington, DC.

**Keywords:** Medical students, information literacy, searching behavior, information seeking, self-directed learning

## Abstract

**Background:**

Medical students must develop self-directed information-seeking skills while they are learning vast amounts of foundational and clinical skills. Students will use different resources for different phases of their training. Information literacy training provided to students will be more impactful when it is embedded into courses or assignments that mimic real-world scenarios. The retention of these skills is also improved by early and frequent instruction sessions, paired with formative feedback from librarian-educators.

**Case Presentation:**

Librarians received student responses to an information literacy question during two cycles of a Grand Rounds activity. Data were analyzed as follows: sources were grouped according to resource type and assessed for quality, and search terms were aggregated and analyzed to determine frequency of use. A librarian-educator presented the compiled data, making suggestions for improving searching and clarifying expectations for how to improve their resource choices for a second Grand Rounds session. Comparing the M2 Grand Rounds case to the M1 case of the same cohort, the frequency of evidence summary and diagnostic tool use increased and the frequency of search engine, textbook/lecture material, and journal article/database use decreased.

**Discussion:**

In the real-world application of back-to-back Georgetown University's Medical Center Grand Rounds exercises, librarian-led instruction on clinical-specific resources appears to be correlated with an improvement in medical students' searching behavior. This trend supports the argument that introducing students early to librarian-led education on clinical-specific resources, and providing feedback on their searches, improves students' information-seeking behavior.

## BACKGROUND

Often, students enter medical school lacking information-seeking skills and yet are expected to be able to use a variety of specialized information resources [1-3]. Medical students are also expected to develop self-directed learning (SDL) skills and habits, including identifying appropriate resources for learning [3-6]. The SDL process, as defined by the Liaison Committee on Medical Education (LCME) standard 6.3, can be summarized in four steps: self-assessment of learning gaps, identification of relevant information, appraisal of information, and instructor feedback [7, 8]. While SDL experiences are considered by the LCME and other accrediting institutions to be integral to medical students' education, educators have received little guidance on how to implement, instruct, and provide feedback on SDL skills, especially in clinical situations [8-10]. Many medical schools have attempted to address the LCME standards by adding case-based learning (CBL) activities to their curricula [11]. CBL activities promote learning by using inquiry-based learning methods, allowing students to apply foundational knowledge to clinical cases, adding meaning to their learning, and deepening their understanding of concepts [5, 12, 13]. CBL outcomes can include clinical problem solving and decision making, diagnostic reasoning, and the information-seeking skills required for the practice of evidence-based medicine (EBM) [12, 14]. Multiple studies suggest that SDL activities, including CBL exercises, should occur often in pre-clinical training curricula, and allow for instructors to correct and redirect students with feedback [11, 12, 14]. Good clinical practice requires both clinical competence and clinical information literacy, therefore, it is highly relevant that as medical education continues to become deliberately learner-centered, educators strive to improve both sets of skills [3, 6, 13].

It is also important to recognize that the types of resources (databases, textbooks, lecture material) that first- or second-year medical students use to find foundational information tend to be different than the types of resources (evidence summaries, Point-of-Care tools) that third- or fourth-year students, residents, or clinicians would use in a clinical context [3, 6]. For example, early in their training, medical students are encouraged to consult evidence summaries, narrative reviews, and textbooks to answer background questions about unfamiliar topics. They search databases to find primary research to help answer foreground questions or to conduct literature reviews. As medical students progress into their clinical years, they use Point-of-Care (PoC) resources such as diagnostic generator tools, drug references, and evidence summaries to get quick answers in clinical settings [6, 15-17]. Proficiency in the use of one type of tool does not necessarily translate into proficiency in another. Choosing the correct resource for the correct context is also a skill that needs to be taught. In a study by Maranda et al., students self-reported that when they were taught how to choose appropriate resources for their information needs, they were more efficient searchers, better able to answer clinical questions, and more able to maintain a positive attitude toward applying EBM principles to patient care [15]. Capitalizing on opportunities to introduce students to the variety of resources at their disposal and teaching students when it is appropriate to use each resource is a strategy that librarian educators should not overlook.

The timing of and the context in which information literacy skills are taught matters. Previous studies have shown that course-integrated (i.e., embedded) instruction that addresses course requirements and allows students to apply learning to real world settings is both motivating for students and effective in improving students' information literacy skills [1, 3, 10]. Many medical schools in the United States have adopted this approach, providing learner-centered, pre-clerkship curricula in the form of problem-based learning (PBL)[18]. In addition to PBL-framed exercises, Kumar and Edwards emphasize that regular refreshers and interactions with librarians help keep information literacy skills fresh [1]. Minchow et al. asserts that medical programs that embed repeated instruction across four years of training are more likely to form patterns of information-seeking behavior that they will be able to carry with them through their careers [3].

This case report is unique in that prior studies have documented the searching behaviors and resource selection of medical students when they are using resources that are aligned with their level of clinical experience [9, 16, 17]. No previous studies have observed the effects on searching behavior and resource selection when clinical resources are introduced to medical students during their pre-clinical years. Other studies have established the benefits of repeated library instruction sessions on database searching skills, but few, if any, have looked at the benefits of training students to use PoC tools for a clinical scenario [3, 6, 10]. Therefore, this preliminary evaluation of an embedded library instruction session for first- and second-year students on clinical-specific resources provides a unique opportunity for medical school educators to see an example of how the early introduction of clinical specific resources might be integrated into their own curricula.

In earlier iterations of the Grand Rounds course described below, course directors and librarians observed that medical students were using a high number of inappropriate, poor-quality resources. The educators wanted to explore strategies to improve the students' resource selection and introduce tools that would help the students in their clinical training years. The primary objective of this case report is to describe the initial efforts of one institution to improve specific information-seeking behaviors (i.e., selection of clinical resources) of medical students during the Medical Student Grand Rounds course completed in their M1 and M2 years of pre-clinical education.

## CASE PRESENTATION

The observations were conducted at Georgetown University's Medical Center (GUMC), which is comprised of 4 graduate-level health sciences colleges Georgetown University School of Medicine (GUSoM), School of Nursing, School of Health, and Biomedical Graduate Education (BGE). GUMC maintains an affiliation with MedStar Georgetown University Hospital (MGUH), a large, urban, teaching hospital and one of the 10 hospital and clinical sites that comprise the large regional health care system, MedStar Health. Dahlgren Memorial Library (DML) serves the students, researchers, and faculty of GUMC, MGUH, and a handful of health sciences focused undergraduate programs. At the time of the study, DML provided access to 5,920 journals, 138 databases, 26 PoC tools, 19 medical apps, and 5,582 e-books.

**Figure 1.**
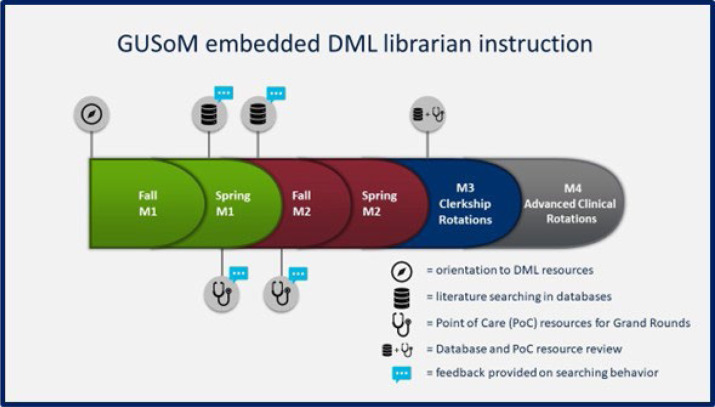
Timing of DML embedded library instruction sessions

DML librarian educators are embedded at multiple points in the GUSoM curriculum (see Figure 1). All incoming GUSoM students are given a library orientation as they enter the program. In the spring of their M1 year, students receive an hour-long instructional session on database searching and the fundamentals of EBM. This instructional session supports a group assignment that requires that students formulate both background and foreground clinical questions and search to find answers to those questions. Initial searches are reviewed by librarian educators and feedback is given to the students on the quality of their search. A similar hour-long session is repeated in the fall of the M2 year. This session is during the early stages of an EBM longitudinal project, in which students must formulate a clinical foreground question relating to treatment, construct a search strategy, and appraise the evidence found. Librarian educators provide feedback during this project about the students' search strategies and article selections. M3 students receive a 30-minute resource and searching review session that supports a required capstone presentation. This presentation is completed during the family medicine clerkship rotation and requires students to apply EBM fundamentals to a clinical case they observe during the rotation. All these sessions focus on database searching and do not provide instruction on resources that are used for diagnostic support in clinical contexts. In contrast, in preparation for the M1 Grand Rounds exercise, a DML librarian gives a 15-minute instructional session on the clinical resources that will best help students solve their case. The tools introduced include diagnostic tools (e.g., DXplain), evidence summaries (e.g., DynaMed and UpToDate), drug information resources (e.g., Micromedex), and eTextbooks (e.g., Harrison's Principles of Internal Medicine). This instruction session focuses on how to use clinical resources to diagnose a patient given limited initial information and patient history.

This case report describes a scenario in which M1 and M2 students are asked to complete a clinically focused exercise, using resources that they may have never heard of, seen, or used before. A few weeks before the case is presented to the students, a DML librarian provides the clinically focused orientation session and explains how evidence summaries and PoC tools will be helpful in providing answers to the Grand Rounds questions. At the start of the Grand Rounds assignment, students are given partial case information that includes a brief description of a patient and a set of symptoms with which the patient presented. Students submit answers to the following questions:

1. What three specific questions related to the patient's [symptom varies by case] do you need answered to better assess the case?
2. What physical exam findings might be important in arriving at a diagnosis and why?
3. What psychosocial issues might be relevant to her history and why? List up to three.
4. Postulate on three potential causes of this constellation of symptoms.
5. What diagnostic tests would you order and why? 6. What sources did you use to answer the above questions? Please list the search terms you used to retrieve information from these sources.

**Figure 2.**
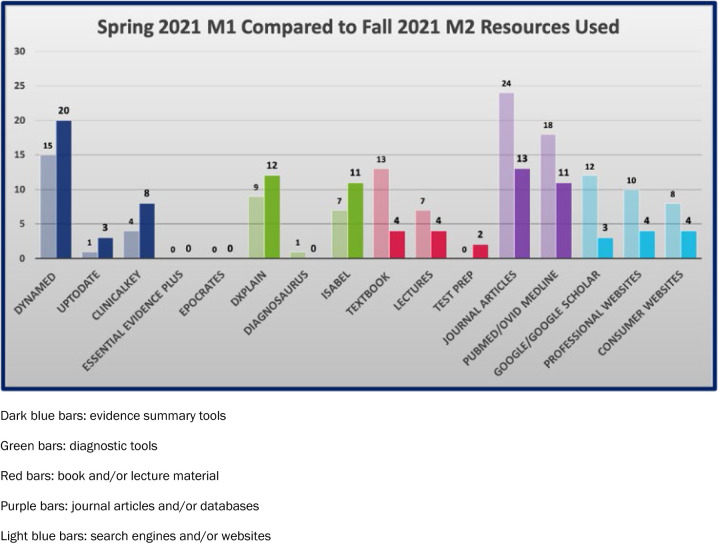
Frequency of resource use in the M1 (translucent) versus M2 (bold) Grand Rounds cases

Question #6 speaks most directly to the impact of the librarian-led instruction session. The DML librarians receive student responses to Question #6 and analyze those responses. First, sources are tallied and grouped according to resource type (e.g., evidence based summaries, diagnostic tools, books/lectures, journals/databases, search engine/website). Next, librarians attempt to assess the quality of certain sources using the Currency, Relevance, Authority, Accuracy, and Purpose, or CRAAP, criteria. Are websites chosen from authoritative sources targeted to health professionals (e.g., American Academy of Family Physicians) or from sources lacking authority and/or targeted at consumers (e.g. WebMD)? Are journal articles relevant to the presented case, reflective of current practices, and published by reputable journals? Third, search terms provided by students are aggregated and analyzed to determine the frequency of use. Finally, a DML librarian educator, as a member of a specialist expert panel, presents the compiled data during the final case presentation, making suggestions for improving searching and clarifying expectations for how to improve their resource choices for their M2 Grand Rounds session. This process is repeated in the fall of the M2 year with a different Grand Rounds scenario. Figure 2 shows the same cohort of medical students, comparing the frequency of sources used between their M1 and M2 Grand Rounds cases. Additional cohort years (not pictured) showed similar trends in resource use. Data analysis is described more fully in the data files that can be found in the OSF data repository [19].

## DISCUSSION

In the M1 Grand Rounds case, students tended to rely heavily on Google and consumer-based websites to find potential diagnoses (see Figure 2). Comparing the M2 Grand Rounds case to the M1 case, the frequency of evidence summary and diagnostic tool use increased, and the frequency of search engine, textbook/lecture material, and journal article/database use decreased. It is not surprising that M1 students turned to search engines and websites for their Grand Rounds exercise. Familiarity and ease of use are important characteristics in students' choice of resources. Multiple studies have demonstrated that students tend to choose resources that are easy to use, freely available, and easily accessible [6, 17, 20, 21]. Google, Google Scholar, and Wikipedia are reported as being frequently used resources [6, 15, 17, 20]. Cognitive load theory may partly explain this commonly observed behavior. Cognitive load theory states that if learning requires a significant amount of mental effort for understanding difficult material, the barriers associated with accessing that material should be reduced to a minimum to optimize learning [18]. Using unfamiliar tools while also trying to assimilate newly acquired foundational and clinical knowledge during the first Grand Rounds scenario likely overtaxed the medical students' cognitive loads. The DML librarian-led instruction reduced the mental burden on medical students by giving them an early introduction to clinical resource tools, in a real-world application. Following this instruction with guided support and feedback reinforced these lessons for the M2 Grand Rounds exercise.

The back-to-back GUSoM Grand Rounds exercises are exemplars of the PBL activities that previous studies have shown to be effective in improving information literacy skills [18]. Between the M1 and M2 Grand Rounds exercises, the students' resource choices skewed more heavily towards the evidence summaries and diagnostic tools. These types of resources are more authoritative and more appropriate for a clinical context, particularly when analyzing a clinical presentation and conducting a differential diagnosis. This trend supports the argument that introducing students early to librarian-led education on clinical-specific resources, and providing feedback on their searches, improves students' information-seeking behavior.

There are limitations to the data that can be extracted from the current iteration of the Grand Rounds assignment. Question #6 as it is written today provides little insight on the pathways that students take to choose their cited sources. Often students list a website URL or journal citation and librarians are unable to determine how they arrived at that source. Did students find their source because of a Google search? Did they launch into a reference linked within an evidence summary or a diagnostic tool? Librarians also are not told whether the students felt that their search terms or sources were successful in helping them answer the other five case questions. Knowing the frequency of search terms, in the absence of a measure of the usefulness of those terms, is not a particularly helpful piece of data. Redesigning Question #6 in order to extract more meaningful data is desirable, but any modifications would need to be carefully thought out. As the main purpose of the Grand Rounds exercise is to introduce medical students to clinical problem solving and decision making and diagnostic reasoning, students should not be asked to spend more time addressing information literacy questions than case-based questions. Therefore, the exercise needs to remain primarily focused on the clinical aspects of the case, and the information literacy questions must continue to play a secondary role in the overall assignment. A stand-alone survey, focusing on information-seeking behaviors, might be an alternative option, but embedding the question into a required assignment guarantees 100% participation, which stand-alone surveys rarely achieve. Ideally, future iterations of Question #6 will be modified, and additional data gathered so that even more meaningful conclusions can be drawn about the impact of library instruction on medical students' information-seeking behavior. Future research might also include an exploration into whether instruction in the use of PoC tools in the foundational years leads to increased usage of or comfort with using PoC tools in the M3/M4 or residency training.

Medical students must develop self-directed information-seeking skills while they are learning vast amounts of foundational and clinical skills. Students may use different resources for different phases of their training. The information literacy training provided to students can be more impactful when it is embedded into courses or assignments that allow students to apply what they are being taught in a real-world scenario. The retention of these skills can also be improved by early and frequent instruction sessions, paired with formative feedback from librarian educators. In the real-world application of back-to-back GUSoM Grand Rounds exercises, librarian-led instruction in the use of clinical-specific resources appears to be correlated with an improvement in medical students' information-seeking skills when comparing their searching behavior between the first and second year of preclinical curricular instruction. Course instructors and librarian educators can consider the following observations as they decide the frequency, timing, and content of the instruction that they provide to medical students.

## Data Availability

Data associated with this article are available in the Open Science Framework repository at https://osf.io/xahtu/.
